# Engineered silica nanoparticles as additives in lubricant oils

**DOI:** 10.1088/1468-6996/16/5/055005

**Published:** 2015-10-16

**Authors:** Teresa Díaz-Faes López, Alfonso Fernández González, Ángel Del Reguero, María Matos, Marta E Díaz-García, Rosana Badía-Laíño

**Affiliations:** 1 Department of Physical and Analytical Chemistry, Faculty of Chemistry, University of Oviedo, Av. Julián Clavería, 8, 33006-Oviedo, Spain; 2 Department of Construction and Manufacturing Engineering, University of Oviedo, Gijón, Spain; 3 Department of Chemical and Environmental Engineering, Faculty of Chemistry, University of Oviedo, Av. Julián Clavería, 8, 33006-Oviedo, Spain

**Keywords:** engineered hybrid SiO_2_ nanoparticles, surface functionalization, dispersion stability, tribological properties, base oil

## Abstract

Silica nanoparticles (SiO_2_ NPs) synthesized by the sol–gel approach were engineered for size and surface properties by grafting hydrophobic chains to prevent their aggregation and facilitate their contact with the phase boundary, thus improving their dispersibility in lubricant base oils. The surface modification was performed by covalent binding of long chain alkyl functionalities using lauric acid and decanoyl chloride to the SiO_2_ NP surface. The hybrid SiO_2_ NPs were characterized by scanning electron microscopy, transmission electron microscopy, Fourier transform infrared spectroscopy, simultaneous differential thermal analysis, nuclear magnetic resonance and dynamic light scattering, while their dispersion in two base oils was studied by static multiple light scattering at low (0.01% w/v) and high (0.50%w/v) concentrations. The nature of the functional layer and the functionalization degree seemed to be directly involved in the stability of the suspensions. The potential use of the functional SiO_2_ NPs as lubricant additives in base oils, specially designed for being used in hydraulic circuits, has been outlined by analyzing the tribological properties of the dispersions. The dendritic structure of the external layer played a key role in the tribological characteristics of the material by reducing the friction coefficient and wear. These nanoparticles reduce drastically the waste of energy in friction processes and are more environmentally friendly than other additives.

## 1. Introduction

Nanoparticles (NPs), particles with at least one dimension between 1 and 100 nm, exhibit specific properties which are not present in the material at a macroscopic scale. These special properties have been widely studied and exploited in several scientific and technological fields, such as, medicine, electronics, optics, magnetic data storage, biotechnology, etc. Dispersing nanometer sized particles in a conventional base fluid results in nanoscale colloidal suspensions, commonly termed ‘nano-fluids’ [1, 2]. When the base fluid is a lubricant oil the term ‘nanolubricant’ is commonly used [3].

The improved tribological properties of nano-lubricants, compared to the base lubricant, are the result of the combination of some features of the NPs. Firstly, due to their high surface area/volume ratio NPs provide a large interfacial surface area to enhance interaction with the surface material of friction pairs forming a protective film, thus reducing friction and wear [4]. As a result of their small size, NPs also provide a mending effect by filling the surface roughness points, forming a thin physical tribofilm during the frictional process that not only bears the load but also separates the rubbing faces [5]. NPs can act as spacers, rolling between the friction surfaces, changing the pure sliding friction to mixed sliding-rolling friction. The abrasiveness of hard NPs may have a polishing effect by reducing the roughness of the rubbing surfaces [6]. Also, NPs may reduce compression stress concentrations associated with high contact pressure by bearing the compressive force depressively. There are a number of excellent reviews in which the synthesis, characterization and applications of nanofluids and nanolubrication are overviewed [7–10].

One of the main challenges to develop a stable nanolubricant formulation is to disperse the NPs in the liquid base oil. Due to their high surface energy, NPs tend to aggregate, cluster or coagulate. Besides, due to the weight of the aggregates formed, they cannot be maintained in suspension by Brownian agitation and tend to sediment. Many studies have been made to prepare stable nanolubricants, so preventing/minimizing those drawbacks. For example, intensive magnetic agitation, ultrasonic agitation and/or any other mechanical stirring are widely used physical techniques for breaking large agglomerates and dispersing particles in the base lubricant. Some other works deal with the electrostatic stabilization (charge repulsion) by which the NPs acquire surface charges through one or more of the following mechanisms: (i) adsorption of ions, (ii) dissociation of surface charged species, (iii) isomorphic substitution of ions, (iv) accumulation or depletion of electrons at the surface, and (v) physical adsorption of charged species onto the surface [11]. Addition of surfactants is also a widely used technique; for example, some metal oxide NPs do not present affinity with the base oil and their dispersion is not easy without the use of surfactants [12]. Ultrasonic dispersion with surfactants is also successful [13].

Long-term stability of nanolubricants can be achieved by use of surfactant-free functionalization by grafting hydrophobic functional groups on the surface of the NPs by chemical routes. In fact, synthesis of hybrid NPs in which the inorganic NPs are surface-modified with organic layers are promising as lubricant additives [14], although their use in the improvement and/or development of new nanolubricants is still in its infancy.

Many types of nanoparticles have been used as lubricant additives, such as organic [15–17] (graphenes, fullerenes, Teflon, graphite, diamond) and inorganic [15, 18–22] (metals, sulfur, fluorides, borates, metal oxides and hydroxides) ones. Among them, the use of surface-modified SiO_2_NPs as lubricant additives is scarcely reported [15, 23–25] in spite of the fact that SiO_2_ NPs possess excellent mechanical properties in terms of hardness, thermal stability and large surface area. Besides, SiO_2_ NPs are cheap, available on the market, and high quality SiO_2_ NPs can even be prepared by sol–gel technology in the lab. In 2006, Li *et al* prepared dispersible SiO_2_ NPs by an *in situ* surface modification approach in which the hydrolysis product of sodium metasilicate was used as the monomer and a silane coupling agent was the long chain terminator (iso-octanol, octylic acid) [26]. According to the authors, these NPs have superior dispersibility and stability in many types of organic solvents and tribological studies also revealed potential in lubrication. More recently, Peng *et al* investigated the tribological properties of liquid paraffin with oleic acid-modified SiO_2_ NPs and found that optimal concentrations of SiO_2_ NPs were associated with better tribological performance than pure paraffin oil [27]. They also reported that the SiO_2_ NP tribological properties depended on the NP size, with those of 58 nm diameter providing better friction-reduction, anti-wear and load-carrying capacity than the pure paraffin oil. In a recent paper, Kim and Archer densely grafted SiO_2_ NPs with amphiphilic organic chains to create a covalently tethered corona of sulfonate-functional alkylaryl chains around the NPs [14]. These NPs dispersed homogeneously in poly-*α*-olefin base oils and the resulting nanolubricant exhibited superior tribological properties in comparison with the pure base oil.

In line with the previous research work as reviewed above, in the present paper, the surface surfactant-free functionalization of SiO_2_ NPs using organic long-chains was addressed and their performance as additives in base lubricant oils was studied. SiO_2_NPs were synthesized by the sol–gel approach and the surface was engineered by grafting long chain alkyl functionalities using lauric acid and decanoyl chloride with the aim of improving their dispersibility in lubricant base oils. The engineered hybrid SiO_2_ NPs were characterized by scanning electron microscopy (SEM), Fourier transform infrared spectroscopy (FTIR), simultaneous differential thermal analysis (SDTA), nuclear magnetic resonance (NMR) and dynamic light scattering (DLS) experiments, while their dispersion in two base oils was studied by static multiple light scattering (MLS) in a Turbiscan Lab^®^ Expert apparatus at low (0.01% w/v) and high (0.50%w/v) concentrations. The potential use of the engineered SiO_2_ NPs as additives in two lubricant oils specially designed for use in hydraulic circuits has been evaluated by checking the tribological properties of the dispersions.

## 2. Experimental details

### 2.1. Materials and methods

#### 2.1.1. Materials

Tetraethylorthosilicate (TEOS) reagent grade 98%, (3-trimethoxysilylpropyl)-diethylenetriamine (TMSDETA) technical grade, aminopropyltriethoxysilane (APTEOS) ≥98%, tetrahydrofuran (THF) anhydrous ≥99.9%, lauric acid (LA) <98%, decanoyl chloride (DCl) 98%, trimethylamine (TEA) 99.5%, 4-(dimethylamino)pyridine (DMAP) >99%, 2-propanol, anhydrous 99.5%, dichloromethane, HPLC grade Chromasolv plus ≥99.9% , and cellulose dialysis sacks (MWCO 12 000) were purchased from Sigma-Aldrich (USA). Ethanol gradient grade for LC was supplied by LiChrosolv (Germany). Ammonium hydroxide 25% was purchased from Panreac (Spain), and toluene of HPLC grade from J T Baker (USA).

Base oils ISO 32 and 68, were kindly provided by REPSOL S.A. (Spain). These type of oils are suitable for hydraulic circuits under moderate working conditions, as well as for all types of machinery where ‘R&O’ (rust and oxidation inhibited) type oils are required. The specifications of these oils are shown in table 1.

**Table 1.** Properties of ‘R&O’ base oils.

Physical properties	Method	BO_32_	BO_68_
ISO Viscosity Grade		32	68
Viscosity at 100 °C (cSt)	ASTM D 445	5.4	8.6
Viscosity at 40 °C (cSt)	ASTM D 445	32	68
Viscosity Index	ASTM D 2270	104	98
Flashpoint (°C)	ASTM D 92	215	235
Pour point (°C)	ASTM D 97	−24	−24
Density, g mL^−1^		0.86	0.89
Oxidation, AN 2500 h (mg KOH g^−1^)	ASTM D 943	2	2

### 2.2. Synthesis and functionalization of SiO_2_ NPs

#### 2.2.1. Synthesis of SiO_2_ NPs

SiO_2_ NPs were synthesized according to the protocol established by Echeverrie and Giraldo [28]. Briefly, 22.5 mL of 25% ammonium hydroxide and 9 mL TEOS were added to 312.5 mL ethanol in a reaction flask under constant stirring (750 rpm). The reaction mixture was kept under reflux at 60 °C for 24 h. Then, the solvent was removed in a rotary evaporator at 45 °C until the sample was almost dry. Finally, the reaction product was heated overnight in an oven at 70 °C to obtain a fine white powder of SiO_2_ NPs. In order to activate the surface of the NPs, a thermal activation was carried out by heating the sample at 130 °C for 21 h and then at 250 °C for 3 h. These final activated SiO_2_ NPs were stored in a desiccator until further use.

#### 2.2.2. Functionalization of SiO_2_ NPs

(a) *Synthesis of amino-functional SiO*_*2*_ *NPs*. Exposed amine groups on the SiO_2_ surface were performed by functionalization with two different silane coupling agents, APTEOS and TMSDETA. An adaptation of the protocol by Kim and Archer was used [14]. One gram of SiO_2_ NPs was suspended in 70 mL water using an ultrasound bath for a few minutes after which 0.1M NaOH solution was added dropwise under constant stirring until pH 9 was reached. The dispersion was warmed up to 95 °C and 1.43 mL TMSDETA or 1.30 mL APTEOS was then added. The mixture was kept at 95 °C until its volume was reduced to 1/3. This process was repeated until a total amount of 4.29 mL TMSDETA or 3.90 mL APTEOS was added. Finally, the suspension was left at 95°C until more than 50% solvent was removed. Once the suspension was cooled down, it was further dialyzed using a MWCO 12 000 dialysis sack versus deionized water for 2 days in order to remove the reagents excess. The final products were precipitated three times using THF to remove completely the remaining water and centrifuged at 7500 rpm for 20 min.

(b) *Synthesis of amino-SiO*_*2*_ *NPs with lauryl pendant groups.* 1 g of the solid obtained in (*a*) was suspended in 180 mL of toluene:2-propanol mixture (5:1, v/v) and stirred overnight to ensure a homogeneous suspension. 3.30 g of LA solubilized in 50 mL of toluene:2-propanol mixture (5:1, v/v) was then added, allowing it to react for 72 h under constant stirring at 1000 rpm at room temperature. The NPs were then separated by centrifugation at 7500 rpm for 20 min and washed three times with toluene:2-propanol mixture (5:1, v/v) by re-suspending and centrifugation. The functional solids, 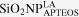 or 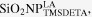 were dried at 40 ± 2 °C overnight and stored in a desiccator until further use. A molar 1:1:3 ratio NPs:APTEOS:LA and NPs:TMSDETA:LA was strictly kept throughout the syntheses.

(c) *Functionalization of amino-SiO*_*2*_*NPs with decanoyl chloride*. A proper amount of SiO_2_NP_APTEOS_ obtained in (*a*) was heated at 45 °C for two hours in an oven in order to completely remove any traces of remaining THF. One gram of solvent-free SiO_2_NP_APTEOS_ was dispersed into 50 mL dichloromethane using an ultrasonic bath and 1.66 mL DCl, 1.67 mL TEA and about 100 milligrams of DMAP as catalyst were then added. The mixture was allowed to react at 45 °C continuously stirring under inert N_2_ atmosphere for 24 h. Then, the solid product was separated by centrifugation at 7500 rpm for 30 min, washed twice with dichloromethane and centrifuged. Finally, the material was washed with ethanol until the supernatant lost its orange color. The functional material 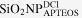 was obtained after centrifugation and dried overnight at 40 ± 2 °C. It was stored in a desiccator until further use. A molar 1:1:3 ratio NPs:APTEOS:DCl was strictly kept throughout the syntheses.

### 2.3. Characterization of the NPs

#### 2.3.1. Morphology and dimensional analysis

The morphology and size of the NPs were checked using a JEOL-6610LV field emission scanning electron microscope (JEOL, Japan) equipped with an integrated INCA Energy 350-Xmax 50 microanalysis system. The microscope was operated at 20 kV. SEM samples were prepared as follows. A few milligrams of solid were suspended in 1 mL ethanol and sonicated for 3 min. Then, three aliquots of 0.1 mL of the homogeneous suspension were dropped on a sample holder and left to evaporate. Samples were coated with a thin gold layer using a high vacuum evaporator Bal-Tec SCD 005 with cold cathode vaporization due to their low electrical conductivity. The mean particle size was analyzed from the digitized images using ImageJ Tool software where at least 250 observations were performed for each sample. The size and morphology of the synthesized nanoparticles were also evaluated by a JEOL JEM-2100F field emission transmission electron microscope, operated at 200 kV. The microscope was equipped with an ultrahigh-resolution pole-piece, which provided a point-resolution better than 0.19 nm, and an energy dispersive x-ray (EDX) detector for elemental microanalysis. Size distribution by number was also determined by DSL using a Zetasizer Nano ZS (Malvern Instruments Ltd, Worcestershire, UK) for the synthesized materials. Samples were taken from each raw synthesis mixture and diluted (1:10 v/v) before being measured at room temperature.

#### 2.3.2. ATR-FTR

Infrared spectra were performed using a Varian 670-IR FTIR spectrometer with a Golden Gate attenuated total reflectance (ATR) device. The samples (dry powder) were put on the ATR crystal and pressed with the anvil to improve the contact between the sample and the device. A resolution of 4 cm^−1^ and 16 scans were used. Spectra were recorded between 600 cm^−1^ and 4000 cm^−1^. Background was taken with the open ATR.

#### 2.3.3. Solid state NMR (SS-NMR)

Solid-state NMR experiments were performed on a Bruker Avance III 400 spectrometer, operated at 400.14 MHz for ^1^H, 100.62 MHz for ^13^C and 79.49 MHz for ^29^Si. All spectra were measured at room temperature. For ^1^H and ^13^C measurements, the magic-angle spinning (MAS) frequency was set at 10 kHz and a commercial 4 mm MAS NMR probe was used. Chemical shift was externally referenced to adamantine. For ^1^H Bloch-decay experiments, the number of scans was set at 128 with a 5 s recycle delay while for ^13^C {^1^H} cross polarization spectra was set at 1000 with 4 s of recycle delay and 5 ms of contact time. The ^29^Si measurements were carried out setting the MAS frequency at 4.5 kHz and using a commercial 7 mm MAS NMR probe. ^29^Si {^1^H} cross polarization spectra (1000 scans) were measured with a recycle delay of 5 s and a contact time of 8 ms. Chemical shift was externally referenced to Q_8_M_8_ (Octakis(trimethylsiloxy)silsesquioxane).

#### 2.3.4. Elemental and thermogravimetric analyses

Elemental analyses (C, N and H) of SiO_2_ NPs were performed on an Elementar GmbH Vario EL analyzer by combustion with pure O_2_ at 1000 °C. Thermogravimetric analysis (TGA) and SDTA were carried out in a Mettler-Toledo TGA/SDTA 851 thermogravimetric analyzer. Thermograms were obtained at a heating rate of 10 °C·min^−1^ from 25 °C to 1000 °C under dynamic air atmosphere (10 mL·min^−1^). In all experiments about 30 mg of powder sample was thermally treated.

### 2.4. Preparation of dispersions

#### 2.4.1. Procedure for dispersing NPs

As the viscosity of the base oils used in this work was not high, we used a probe ultrasonic method for dispersing NPs into the base oils at different concentrations. Briefly, a proper amount of hybrid NPs was added into a beaker containing 40 mL base oil. The mixture was sonicated for 15 min using a Ti probe in pulsed mode (sonication/pause rate 3/3 s) at 500 W, 50% amplitude and 20 kHz frequency. During sonication, the beaker was kept in a cooling water bath at 15 °C in order to avoid the sample heating.

#### 2.4.2. Dispersion stability study

The stability of the suspensions was determined by static multiple light scattering (MLS) in a Turbiscan Lab^®^ Expert apparatus (Formulaction, France) provided with an Ageing Station (Formulaction, France). MLS consists of sending a light beam through a cylindrical glass cell containing the sample. The light source is an electro luminescent diode in the near infrared (NIR, *λ* = 880 nm). 20 mL samples were placed in the cylindrical glass test cells and two synchronous detectors received the light transmitted (TS) through the sample (180° from the incident light), and the light backscattered (BS) by the NPs in the sample (45° from the incident light). The optical reading head scans the sample in the cell, providing BS and TS data in % relative to standards (suspension of monodisperse spheres and silicone oil) as a function of the sample height (in mm). Backscattered and transmitted light was monitored as a function of time and cell height for 8 days, every 2 h, at 33 °C. These profiles build up a macroscopic fingerprint of the sample at a given time, providing useful information about changes in size distribution or appearance of a creaming layer or a clarification front with time [29–31].

#### 2.4.3. Tribological properties

Tribological properties were evaluated under extreme load conditions as specified in the ASTM D2783 standard using a Stanhope Seta Shell Four-Ball E.P. Lubricant Tester working at 1470 rpm shaft speed. 12.7 mm diameter tests balls with 0.035 *μ*m roughness were made from AISI 52100 steel (hardness 65 RC; 0.98–1.1%C, 0.15–0.30%Si, 0.25–0.45%Mn, 1.30–1.60%Cr, <0.025%P, <0.025%S). The assay consisted in a rotating steel ball under load against three stationary steel balls, held in a cradle shape and immersed in the tested nano-dispersion. After finishing the assay, the wear scars in the stationary balls were evaluated with a Nikon PFX optical microscope coupled to a Nikon F-301 CCD. Repeatability and reproducibility were verified following ASTM D2783 recommendations. All test-section components were ultrasonically cleaned in heptane for 3 min, then rinsed with ethanol and dried under hot air before and after tests.

## 3. Results and discussion

As a general rule, metal oxide NP suspensions in hydrophobic media tend to have a poor stability, especially when the concentration rises. This is due to aggregation and sedimentation phenomena which are related to the presence of hydroxyl groups on the SiO_2_ NPs surface, their large specific area and specific surface energy. The higher the amount of surface hydroxyl groups, the lower the hydrophobicity of the NP and, therefore, the lower stability in hydrophobic media. A straightforward method to improve the compatibility of SiO_2_NPs with oily matrices, thus improving their dispersibility, is grafting hydrophobic chains to SiO_2_ NPs surface.

**Figure 1.**
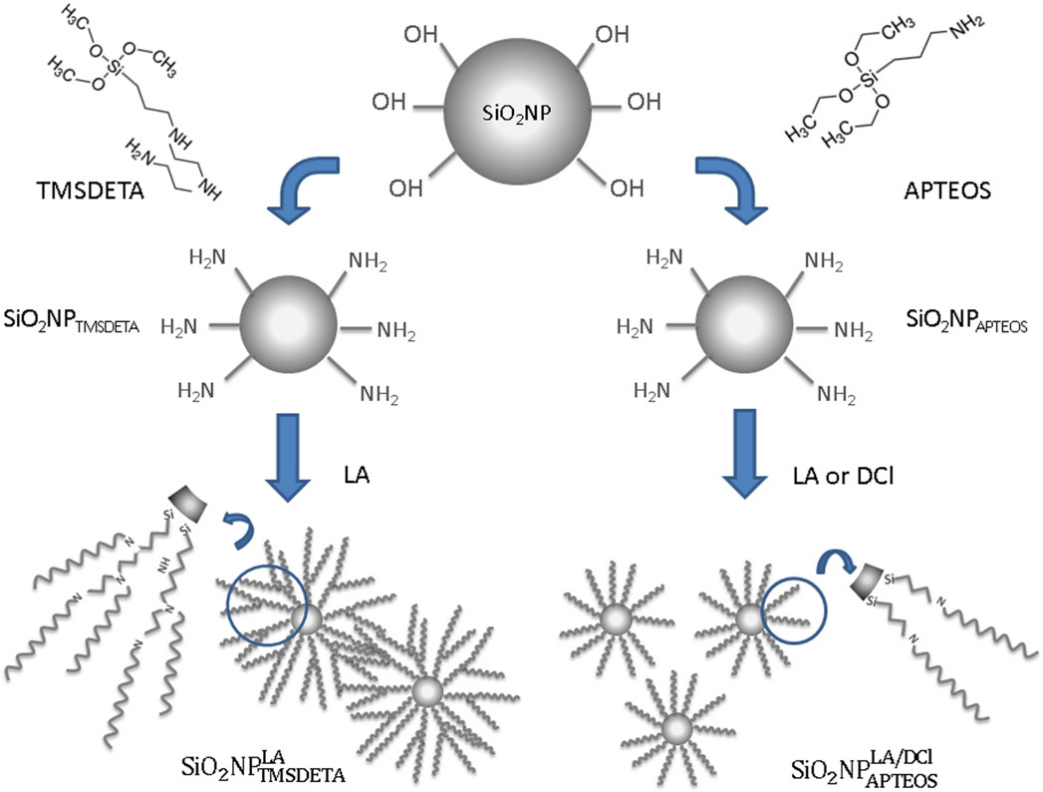
Sketch of the hybrid SiO_2_ NPs functionalization.

In the present research, surface modification of SiO_2_ NPs was addressed by using two silane coupling agents which provide amine groups for further reaction (figure 1). These silane coupling agents, TMSDETA and APTEOS (see figure S1), bound to the SiO_2_ NP surface forming 1–3 Si–O–Si links through a condensation reaction with the surface silanol groups. The presence of the amine groups allowed for facile linker chemistry with LA and DCl (see figure S1), which should provide the hydrophobic shell (hybrid SiO_2_ NPs) to improve the NP stability in hydrophobic media.

**Figure 2.**
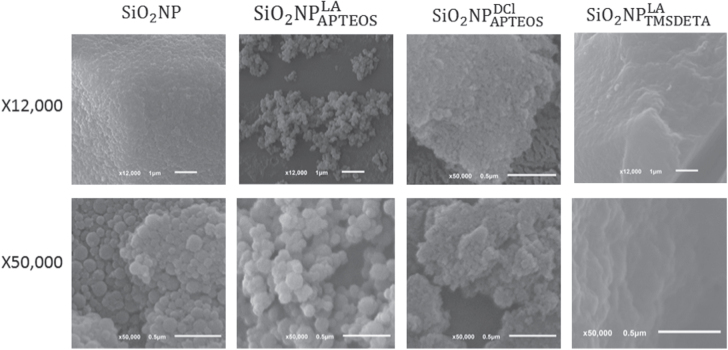
SEM images of the synthesized NPs.

SEM, high-resolution transmission electron microscopy (HR-TEM) and DLS techniques were used in order to study the shape, size and morphology of raw SiO_2_ NPs, as well as to check possible changes after the surface modification steps. SEM and HR-TEM images revealed monodisperse spherical SiO_2_ NPs with a mean diameter 102 ± 33 nm (figures 2, S2 and S3(a)). This value was slightly smaller than that obtained by DLS, 115 ± 36 nm. This may be due to the fact that DLS provided the diameter of the solvated NPs [32]. Also, SEM and HR-TEM images of the hybrid SiO_2_ NPs showed that only 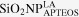 kept a similar morphology to that of raw NPs: monodisperse spherical NPs with a diameter of 139 ± 31 nm (figures S2 and S3(b)). The remaining hybrid NPs had substantially different morphology from that of the raw SiO_2_ NPs. In fact, 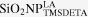 showed a compact laminar structure with sharp edges suggesting a key effect of the coupling agent on the morphology. The differences in morphology observed between 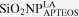 and 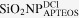 must be related to the synthesis procedure. In the latter, the solvents, temperature and presence of a catalyst should be responsible for the fluffy morphology and NP agglomeration (figure 2). Also, the morphology of the hybrid NPs, 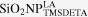 and 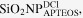 is not clearly evident from HR-TEM images (figure S2) because the nanoparticles are strongly aggregated (thanks to the hydrophobic surface shell) during the drying on the support film of the HR-TEM specimen grid. However, as will be shown below, the dispersibility of 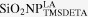 in the base oil was high.

Table 2 summarizes the C, N and H elemental composition of the synthesized NPs. As can be observed, hybrid APTEOS-based NPs had a higher nitrogen content than hybrid-TMSDETA-based ones. These results may be surprising, taking into account that TMSDETA has three nitrogen atoms whereas APTEOS contains only one. However, the presence of three nitrogen atoms in TMSDETA may allow further functionalization with LA or DCl. In fact, a comparison of the carbon:nitrogen (C:N) experimental atomic ratio with the expected one, considering a reaction yield of 100%, the efficiency of the LA and DCl functionalization, was about 40% in the case of APTEOS-based SiO2 NPs while for 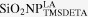 the efficiency was about 80% if one LA was considered, 48% for two LA molecules and 35% for three LA groups. In light of these results, it is suggested that a more hydrophobic branched shell has been created around the 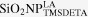.

**Table 2.** Elemental analyses of the synthesized NPs.

	Elemental analysis % weight			Atomic ratio
Nanoparticle	**N**	**C**	**H**	**C:N**
SiO_2_NP	0.2	2.07	1.0	—

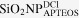	4.7	22.7	4.9	11:2

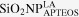	5.1	36.6	7.1	17:2

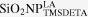	3.4	14.7	3.5	10:2


The different steps of the synthesis and functionalization of bare SiO_2_ NPs were followed using ATR-FTIR and SS-NMR Infrared spectra of the SiO_2_NPs; 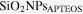 and 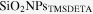 clearly showed the bands of the SiO_2_ structure of the NPs [33] as well as bands belonging to the organic functional groups involved in the coupling reactions. An intense band between 1020 and 1060 cm^−1^ from the Si–O–Si (siloxane), the asymmetric stretching band from Si–O, a shoulder at 952 cm^−1^ from Si–OH stretching and a band near 780 cm^−1^ from Si–O vibrations are typical for a SiO_2_ xero-gel structure [34]. Furthermore, the broad band around 3200 cm^−1^ from N–H (or NH_2_) asymmetric and symmetric stretching, a band at 1540 cm^−1^ from the –NH_3_^+^ symmetric flexion vibration, a N–H flexion vibration band at 670 cm^−1^, together with weak bands at 2915 cm^−1^ and 2845 cm^−1^ from –CH and –CH– stretching modes and weak bands at 1454 cm^−1^ and 1396 cm^−1^ from CH_3_– and –CH_2_– deformations, confirmed the presence of the amine-based coupling agents. Upon amide formation with LA and DCl (see figure S4), the intensity of the bands belonging to CH bonds (2915, 2845, 1454 and 1396 cm^−1^) became more intense and well defined. Additionally, in hybrid nanoparticles new bands near 1720 cm^−1^ and 1600 cm^−1^ (1730 cm^−1^ and 1643 cm^−1^ for 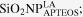 1706 cm^−1^ and 1620 cm^−1^ for 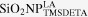 and 1606 cm^−1^ for 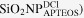 appeared due to the presence of C=O bonds [33]. These results indicated the successful grafting of the hydrophobic tails on the SiO_2_ NPs.

**Figure 3.**
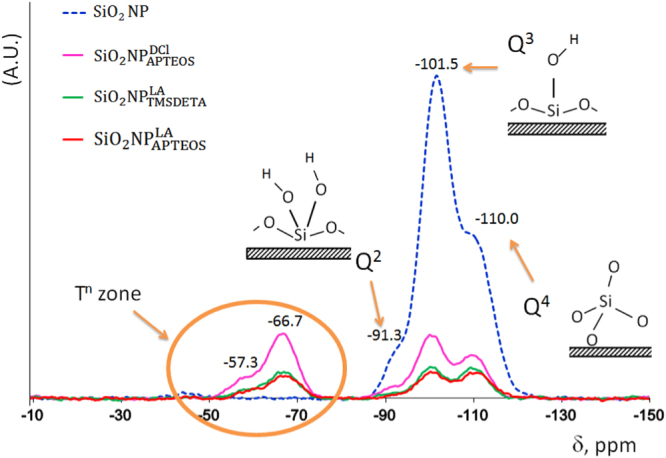
^29^Si solid-state NMR spectra for NPs.

^13^C and ^29^Si solid-state NMR spectra were also evaluated in order to obtain information regarding cross-linking, polymerization and functionalization of the different materials. The^13^C SS-NMR spectra of bare SiO_2_ NPs (figure S5), no signals around 19 ppm or 60 ppm (ascribed to surface etoxy- groups, Si–O–CH_2_–CH_3_), could be detected as should be expected for NPs synthesized using TEOS, which confirmed that the precursor was fully hydrolyzed during the synthesis of the NPs [35]. On the other hand, ^13^C SS-NMR of the hybrid NPs revealed the presence of signals characteristic of Si–CH_2_–CH_2_–CH_2_–N– (between 32.3 and 9.8 ppm) and CH_3_–(CH_2_)*_n_*–CO- (*n* = 8, 10, between 47.7 and 12.0 ppm) structures, due to the amine functional group and the amide formation with the long chain LA (or DCl), respectively (see figure S5). ^29^Si SS-NMR spectra of raw SiO_2_ NPs also showed typical peaks assignable to a Si–O bond: signals at −91.3 ppm, −101.5 ppm and −110.0 ppm that correspond to *Q^n^* shifts (figure 3). The intense signal located at −101.5 ppm (*Q*^3^), is typical for Si atoms bond to a hydroxyl group and three siloxane bridges in a highly cross-linked matrix [36, 37]. After functionalization, new signals appeared in the *T^n^* zone [34] at −57.3 ppm and −66.7 ppm, which confirmed the incorporation of amino groups provided by the silane coupling agents to Si atoms in the surface structure of the xero-gel.

TGA and SDTA showed two different regions for the synthesized materials (figure S6). In the first one, between 100 °C and 350 °C, all the materials exhibited a similar loss of mass (3.7%–5.2%), which can be attributed to a loss of physically adsorbed water (maximum around 100 °C) and residual organic solvents. For unmodified SiO_2_, in the temperature range from 100 to 350 °C, the weight loss was low (<2%). In the second region, between 300 °C and 800 °C, bare SiO_2_NPs remained more or less non affected (loss ∼6% mass) and the TGA curve was practically flat. According to literature, the temperature range from 200 to 400 °C corresponds to surface silanol condensation whereas isolated hydroxyl groups (inside the mesoporous structure) are condensed at higher temperatures (up to 500 °C)[38, 39]. On the other hand, the hybrid NPs suffered the highest loss of mass (figure S6) between 350 and 800 °C. This loss may be attributed to the thermal decomposition of chemically bonded LA and DCl groups. The weight loss for the 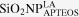 was substantially lower (approximately 25%) than that of 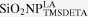 (about a 58%). The hydrophobic grafting (%) was determined by TGA and calculated by equation (1) [40]:

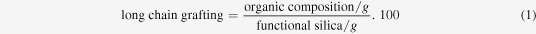
where the amount of organic composition introduced by LA and DCl functionalization was calculated from the TGA weight loss data obtained between 300 and 800 °C. Functional SiO_2_ was the weight retention by TGA corresponding to the bare amino-functional silica at 800 °C. Results are summarized in table 3.

**Table 3.** Thermogravimetric analysis of the synthesized NPs.

Nanoparticles	Weight loss (%), 100–350 °C	Weight loss (%), 350–800 °C	Long chain grafting (%)
SiO_2_NP	2.1	4.4	—

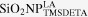	9.0	46	50.5

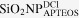	9.0	31	34

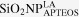	6.4	17	19


These results demonstrate that the grafting degree of 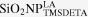 was higher than that of amine-NPs based on APTEOS and are supported by the elemental analysis (*vide supra*) from which the efficiency of the grafting was found to be higher for the SiO_2_ NPs based on the TMSDETA amine-coupling agent.

As stated before, the main objective of the functionalization process was to improve the dispersibility of the NPs used as lubricant additives. With this aim, the two base oils BO_32_ and BO_68_ (table 1), with high stability and specially designed for being used in hydraulic circuits, were selected as dispersion media in order to check the dispersibility of the synthesized NPs. Suspensions of 0.01%, 0.04%, 0.15% and 0.50% (w/v) were prepared according to the protocol described in the experimental section. Changes in the backscattered light (ΔBS) radiation as a function of time and cell height were measured 8 days after the corresponding suspension was prepared. These changes were evaluated by subtracting the initial value of the transmitted (TS) or backscattered (BS) light at *t* = 0 (TS_*t*=0_ or BS_*t*=0_) from the values obtained at a time *t* = *m*, (TS*_t=m_* or BS*_t=m_*) at a given cell height. The value of the backscattered radiation is related to the photon transport mean free path *l*∗, which represents the mean free path run by the photons before losing the direction of the incident light. Considering a narrow area with a differential thickness, d*h*, like that in the measuring system, the BS can be expressed as [41]:

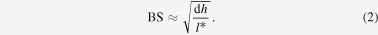
The ΔBS parameter was evaluated in three different height positions of the cell. In the middle part, BS variations involve changes in particle size, whereas ΔBS and ΔTS in the upper and lower part of the cell provide information about migration, sedimentation and creaming phenomena [42]. Increase or decrease of BS with time is related to the ratio particle size/wavelength of incident light, so that when a light source of 880 nm is used, BS grows as the size of the NPs aggregate up to 0.6 *μ*m. If the nanoparticle ‘flakes’ overtake this threshold, BS starts to diminish [43].

According to the experimental protocol used, there was a gap time between finishing the suspension of the NPs using ultrasound and starting the BS measurements. Thus, those unstable suspensions settled before being able to start the measurement (*t* = 0). In these cases, data of ΔBS did not reflect accurately the real processes that occurred in the cell. This fact was particularly evident for SiO_2_ NPs suspensions, where a white layer of settled NPs could be observed in the lower part of the cell by the naked eye before starting measurements. In contrast, this was not observed for hybrid NPs. For this reason, we missed out the discussion of SiO_2_ NP results as they were not comparable to the data obtained for the functional materials.

**Figure 4.**
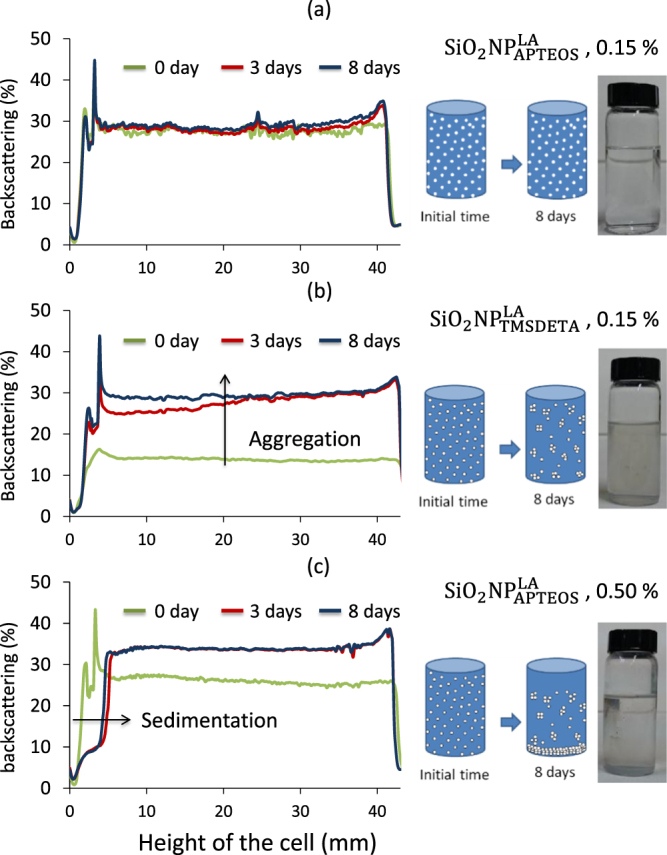
Backscattering (BS) profiles for stable (a), aggregated (b) or sedimented (c) NP suspensions.

Figure 4 summarizes the BS profiles versus the cell height at three different times of the suspension preparation. Figure 4(a) shows the BS profile of a 0.15% (w/v) suspension of 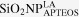 in BO_32_ in which no significant changes in BS were observed even 8 days after its preparation. In contrast, figure 4(b) shows an increased BS with time for a 0.15% (w/v) suspension of 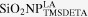 in BO_32_, which was indicative of changes in the NP size due to aggregation. These results suggested that the dendrimer nature of 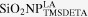 coating favored the self-assembling (aggregation) of the NPs via hydrophobic interactions among the alkane chains. Finally, figure 4(c) shows that an increase in the suspension concentration of 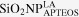 in BO_32_ resulted not only in a BS increase due to aggregation, but also in a sedimentation process as observed for the BS decrease at the bottom of the cell (<5 mm height).

**Figure 5.**
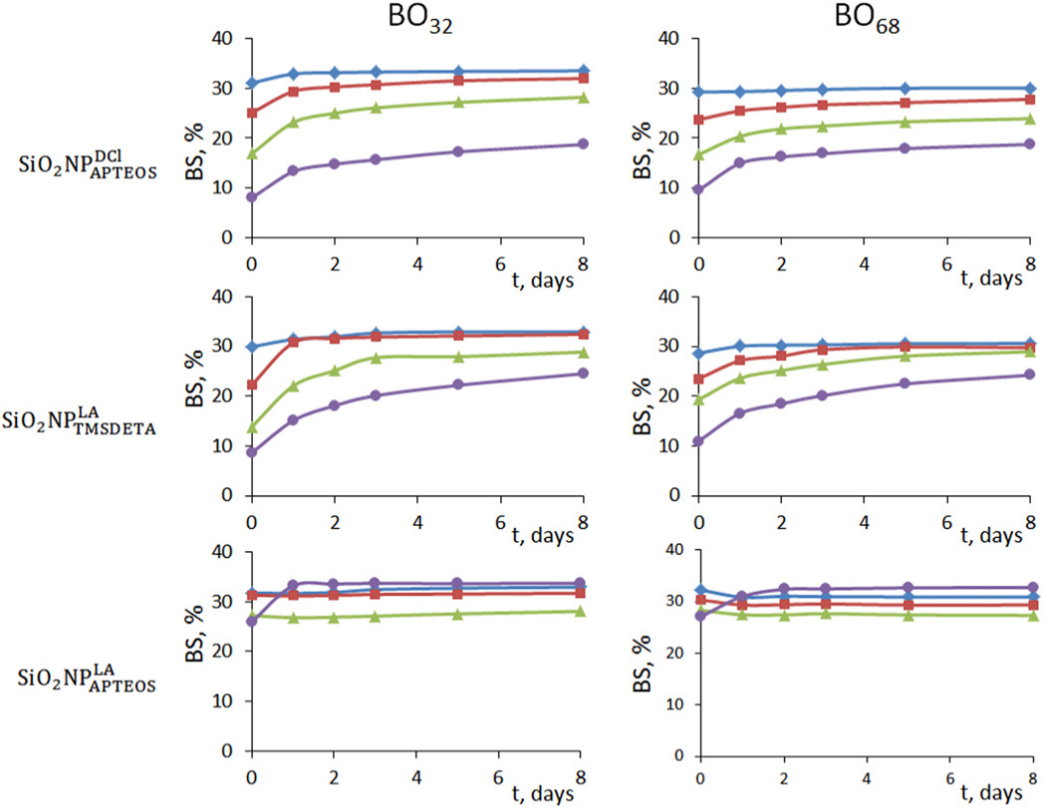
Kinetic BS profiles in the middle zone of the cell (from 10 to 30 mm) for the different hybrid nanoparticles and base oils at 0.01%, 0.04%, 0.15% and 0.50% w/v concentrations.

In figure 5, the BS results obtained for the different hybrid NPs in the two base oils are represented as a function of elapsed time after dispersions preparation and their concentration. In general, it was observed an increase of BS with time along the cell height whatever the base oil used. In most cases, after the second day, BS reached a plateau value that remained constant for at least 8 days. Also, as more diluted the suspensions, the lesser change in BS with time and cell height. The kinetic BS profiles were obtained plotting incremental values of BS (ΔBS) versus time for 8 days. Table 4 summarizes the maximum ΔBS (ΔBS_max_) values obtained by applying equation (3). Measurements were taken in the middle zone of the cell (from 10 to 30 mm) and estimated according to the formula:

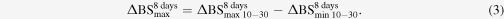
Turbiscan stability index (TSI, equation (4)) was also calculated by the turbidimeter software based on a scan-to-scan difference of the intensity light with time through the complete cell height (H), for every nanoparticle size and/or suspension concentration.

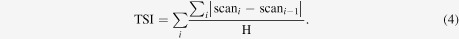
From the data in table 4, it is clear that both the 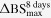 and the TSI were more important in BO_32_, with a lower viscosity than in the more viscous BO_68_. The alkane NPs that exhibited better dispersibility in both base oils in a wide range of concentrations (0.01%–0.15% w/v) were 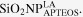 For higher concentrations (e.g. 0.5% w/v), NPs aggregated during the first 48 h increasing the BS variations up to 10%, after which it remained constant (figure 4).

**Table 4.** Maximum backscattering variation, 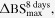 and Turbiscan stability index (TSI) in the middle zone of the cell (from 10 to 30 mm) for an 8-day period.

Silica sample	% w/v	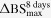 (%), BO_32_	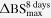 (%), BO_68_	TSI BO_32_	TSI BO_68_
	0.01	2.1	1.57	9.2	9.1
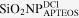	0.04	6.7	4.0	17.0	11.9
	0.15	11.5	7.6	45.0	31.0
	0.50	10.8	9.3	43.0	42.5
	0.01	1.1	1.2	1.8	2.9
	0.04	0.5	0.9	0.9	1.8
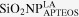	0.15	1.2	0.8	5.0	1.6
	0.50	7.6	5.7	19.5	16.0
	0.01	2.7	2.2	6.5	5.4
	0.04	9.6	5.8	27.0	15.1
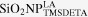	0.15	15.2	9.7	62.0	26.5
	0.50	16.6	13.4	54.0	49.0

From these data, it was clear that not only does the amine silane coupling agent for further functionalization of SiO_2_ NPs have a decisive effect on the stability of the suspensions, but so does the synthesis procedure and physical properties of the base oil. So, when comparing the data (table 3) for 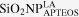 and 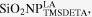 the former exhibited a better dispersibility in both base oils than the latter. As stated before, a possible explanation for these results may take into account the dendrimer-like structure of the 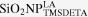 layer.

**Figure 6.**
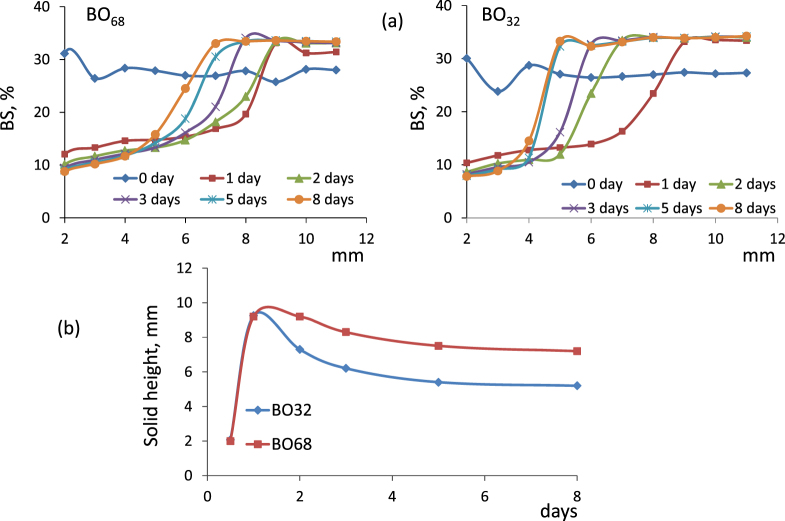
Backscattering (BS) variation at the bottom of the cell from 2 to 12 mm (a) and evolution of the height of the sedimentation front with time (b) for 0.5% w/v 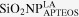 suspension.

In order to shed light on the migration of NPs from the bulk dispersion towards the bottom of the cell, the concentrated 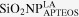 0.5% (w/v) dispersion was used. In figure 6(a) the % BS vs cell height (2–12 mm), after different days of dispersion preparation, was plotted for both base oils. As can be seen, the behavior of these systems does not follow the Mie theory [41], which explains the classical sedimentation phenomenon: BS increase in the lower part of the cell as a consequence of the increment in the concentration of the dispersed phase (sediment) and a simultaneous decrease in the upper part of the cell as a consequence of the clarification phenomenon.

Our system showed atypical behavior, since BS decreased in the lower part of the cell while it increased in the upper part of the cell. After 1 day of preparation, BS decreased in the height range 2–8 mm and that decrease shifted to about 5 mm and 7 mm height after 5 days of dispersion aging in BO_32_ and BO_68_, respectively. At the bottom of the cell, when the concentration is increased and a ‘cake’ of NPs is formed, it is possible to have dependent diffusion leading to a decrease of BS, which is a characteristic phenomenon of particle packing. This trend was also observed in figure 4(c).

According to data in figure 6(a), we can assume that after a consolidation period of about 5 days, three zones may be considered: a zone of NPs with similar size free in solution and a sediment layer, separated by a diffusion interface (≤1 mm). figure 6(b) shows the rate of NP settling by observing the change in height of the sediment layer with time in both base oils. It can be seen that the rate of sedimentation decrease in the more viscous oil BS_68_ as expected due to increased interactions (friction) of NPs with the oil which, in turn, increase the oil dispersion viscosity [44].

## 4. Outlook of engineered hybrid NPs use as lubricant additives

Once the objective of the stability was achieved, the tribological properties of the hybrid NPs were evaluated. The friction coefficient and the average diameter on the wear scar were calculated in a standard 4-ball ASTM 4172-94 assay using hybrid NPs suspended in both BO_32_ and BO_68_ base oils at 0.5% w/v as lubricant. The suspensions were prepared just before starting the tribological experiment so that the aggregation or precipitation effect could be neglected (ΔBS < 3% within the first six hours for cell-heights 10–30 mm).

**Figure 7.**
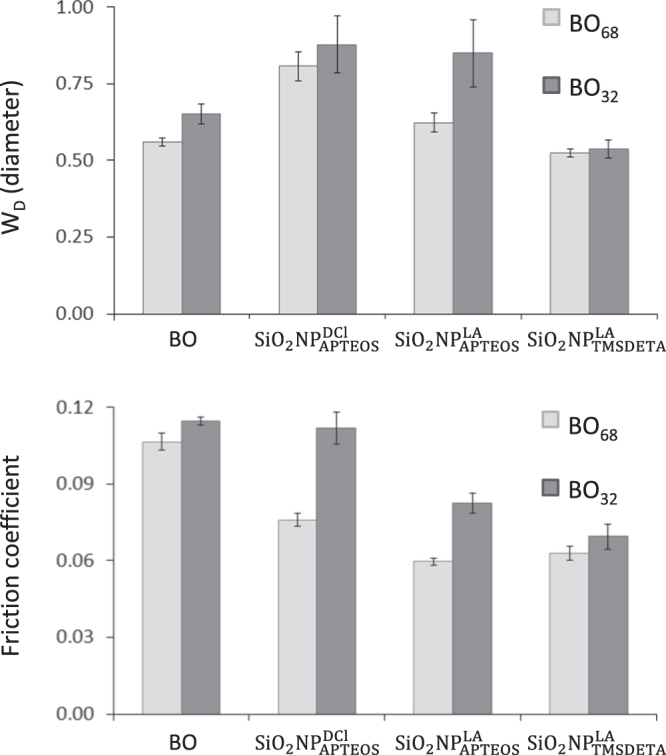
Tribological properties of the different synthesized NPs suspended at 0.5% w/v in BO_32_ and BO_68_.

The results demonstrated that the diameter of the wear scar was larger when NPs were incorporated either the base oil used, except in the case of 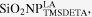 for which the wear scar diameter was similar to that observed for the neat base oil (figure 7). These results could be explained by taking into account the functional surface of the NPs (figure 1). The dendritic-like structure of the cover shell of 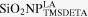 prevents the NP direct metal surface contact, although filling the asperity valleys of the contact surfaces due to their nanosize dimensions was still possible. In the case of 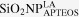 and 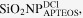 the absence of alkane brushes on the NP surface may allow contact of the NP core with the surfaces, thus summing up their scar effect to that of the base oil. On the other hand, tribological tests also showed that NPs exhibited good friction-reducing properties, up to 40% depending on the NPs and the base oil. So, for BO_68_, hybrid NPs contribute to decrease the friction coefficient, this effect being more important for 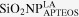 nanoparticles. On the other hand, only those LA-functional NPs improved the friction coefficient at a similar level than the BO_32_ (figure 7).

These data suggested that the alkane functional coating of the SiO_2_ NPs may act as a protective buffer between the contact area, smoothing the rough areas and reducing the friction under shear forces through a rolling mechanism.

## 5. Conclusions

SiO_2_ NPs have been engineered with hydrophobic layers in order to prepare stable suspensions in base oil matrices for their potential use as anti-wear and friction reducing additives. Long alkane chain-hybrid SiO_2_ NPs have been demonstrated to reduce the friction coefficient and wear. This reduction implies a strong reduction in the spend of energy as a consequence of the friction processes, thus also reducing the cost of lubrication. The nature of the functional layer and the functionalization degree seemed to be directly involved in the stability of the suspensions. The dendritic structure of the external layer played a key role in the tribological characteristics of the material. Furthermore, the physical characteristics of the base oil also have an important influence on the anti-wear properties of the NPs. Future work will include the tribological performance of these materials in a formulated oil.
